# Acquisition of a novel conjugative multidrug‐resistant hypervirulent plasmid leads to hypervirulence in clinical carbapenem‐resistant *Klebsiella pneumoniae* strains

**DOI:** 10.1002/mlf2.12086

**Published:** 2023-09-28

**Authors:** Gong Li, Ling Jia, Lei Wan, Lijuan Xia, Ang Gao, Runshi Yang, Ruanyang Sun, Minge Wang, Juan Du, Xinlei Lian, Rongmin Zhang, Liangxing Fang, Xiaoping Liao, Yahong Liu, Bao‐Tao Liu, Jian Sun

**Affiliations:** ^1^ State Key Laboratory for Animal Disease Control and Prevention South China Agricultural University Guangzhou China; ^2^ Guangdong Provincial Key Laboratory of Veterinary Pharmaceutics Development and Safety Evaluation South China Agricultural University Guangzhou China; ^3^ Department of Pathogen Biology and Microbiology Zhejiang University School of Medicine Hangzhou China; ^4^ College of Veterinary Medicine Qingdao Agricultural University Qingdao China

**Keywords:** conjugative virulence plasmid, CR‐HvKP, hybrid plasmid, *Klebsiella pneumoniae*, ST23

## Abstract

The co‐occurrence of plasmid‐mediated multidrug resistance and hypervirulence in epidemic carbapenem‐resistant *Klebsiella pneumoniae* has emerged as a global public health issue. In this study, an ST23 carbapenem‐resistant hypervirulent *K. pneumoniae* (CR‐HvKP) strain VH1‐2 was identified from cucumber in China and harbored a novel hybrid plasmid pVH1‐2‐VIR. The plasmid pVH1‐2‐VIR carrying both virulence and multidrug‐resistance (MDR) genes was likely generated through the recombination of a virulence plasmid and an IncFIIK conjugative MDR plasmid in clinical ST23 18622 isolated from a sputum sample. The plasmid pVH1‐2‐VIR exhibited the capacity for transfer to the clinical ST11 carbapenem‐resistant *K. pneumoniae* (CRKP) strain via conjugation assay. Acquisition of pVH1‐2‐VIR plasmid directly converted a CRKP into CR‐HvKP strain characterized by hypermucoviscosity, heightened virulence for *Galleria mellonella* larvae, and increased colonization ability in the mouse intestine. The emergence of such a hybrid plasmid may expedite the spread of CR‐HvKP strains, posing a significant risk to human health.

## Impact statement

An ST23 carbapenem‐resistant and hypervirulent *K. pneumoniae* (CR‐HvKP) strain VH1‐2 was detected from cucumber in China. VH1‐2 was observed to possess a hybrid virulence plasmid, pVH1‐2‐VIR, which may have arisen from the recombination of a virulence plasmid and an IncFIIK conjugative MDR plasmid in clinical ST23 18622 from a sputum sample. The hypervirulent plasmid pVH1‐2‐VIR was proficient in conjugative transfer to clinical ST11 CRKP, leading to an escalation in virulence and colonization levels. The appearance of such plasmids can be worrisome, which necessitates vigilant observation of their prevalence in the food chain.

## INTRODUCTION

*Klebsiella pneumoniae* is a significant constituent of the human gut microbiota, yet it also serves as a prominent causative agent of invasive nosocomial infections including pyogenic liver abscesses that pose a substantial clinical treatment across the globe, particularly in Asia^1^. Clinical *K. pneumoniae* strains have undergone a transformation into hypervirulent *K. pneumoniae* (HvKP) (initially detected in Taiwan, China in 2002) and carbapenem‐resistant *K. pneumoniae* (CRKP), both of which are currently prevalent in Asia^2^, ^3^, ^4^. Infection by HvKP strains is the cause of significant life‐threatening liver abscesses or pneumonia thereby resulting in a high morbidity and mortality^5^. CRKP strains are responsible for the majority of (70%–90%) clinical carbapenem‐resistant *Enterobacteriaceae* (CRE) infections in several countries including China^6^. This has resulted in untreatable or difficult‐to‐treat infections and poses a pressing public health concern globally. Fortunately, most HvKP strains as of now are susceptible to antibiotic therapy.

A significant threat to animal and human health is the acquisition of a carbapenemase‐encoding plasmid by an HvKP strain, which results in a carbapenem‐resistant HvKP (CR‐HvKP)^7^, ^8^. A fatal outbreak caused by ST11 CR‐HvKP strains carrying a pLVPK‐like hypervirulence plasmid was reported in China^3^ and subsequent CR‐HvKP infections have been documented^9^, ^10^. It was recently discovered that the integration of carbapenemase gene *bla*_KPC‐2_ into an HvKP virulence plasmid, along with the capsular polysaccharide (CPS) regulator *rmpA2*, had resulted in the emergence of hyper‐resistance and hyper‐virulence phenotypes^8^. Fortunately, this plasmid was determined to be nontransferable even though it was mobilizable. A recent discovery in China revealed the insertion of a transposon element carrying*bla*_CTX‐M‐24_ into a pLVPK‐like hypervirulence plasmid in an ST23 CR‐HvKP^11^. However, the nonconjugative characteristic of pLVPK‐like virulence plasmids has constrained the dissemination levels of CR‐HvKP. The greatest concern, however, is a hybrid virulence plasmid that is conjugative. Moreover, composite pLVPK and IncFIB plasmid has been detected in a clinical *Klebsiella variicola* isolate, suggesting a potential for increased dissemination of CR‐HvKP^12^. All previously reported CR‐HvKP strains have been isolated solely from hospitalized patients with no instances from animals, environment, or retail food.

The current safety status of the food chain has attracted significant public attention due to contamination risks and its potential to act as a reservoir for antibiotic resistance genes (ARGs). Numerous studies have reported a high prevalence of antibiotic‐resistant bacteria in the global food chain, particularly in retail^13^, ^14^, ^15^. CRE are also frequently detected in retail meat with some new isolates carrying both *bla*_NDM_ type carbapenemases and the mobilized colistin resistance gene *mcr‐1* ^16^. The safety of fresh vegetables has also become a growing concern due to the rising number of foodborne disease outbreaks linked to contaminated vegetables. These infections are often caused by bacteria exhibiting hyper‐resistance and hyper‐virulence phenotypes, such as Shiga toxin‐producing *Escherichia coli* (STEC), which encoded ESBL in sprouts in Germany in 2011^17^. In our previous study, two CRKP isolates were found in ready‐to‐eat vegetables but the virulence potential of these isolates is currently unknown^18^.

The current study presents novel findings on the identification of a conjugative virulence plasmid (undergone hybridization) with a conjugative IncFII MDR plasmid (isolated from cucumber). Acquisition of this virulence plasmid was observed to understand the level of virulence and intestinal colonization.

## RESULTS

### Genome sequence analysis of *K. pneumoniae* strains VH1‐2 and 18622

Between May and November 2017, the CR‐HvKP ST23 strains 18622 and VH1‐2 were obtained from an inpatient sputum sample and cucumber, respectively, in the Qingdao region of Shandong province, China. These strains exhibited highly similar pulsed‐field gel electrophoresis (PFGE) patterns (Figure S1). ARG and virulence gene analysis indicated a significant degree of relatedness between strain 18622 and VH1‐2 (Table S1). Altogether, these findings indicate that VH1‐2 and 18622 are phylogenetically closely related.

**Figure 1:**
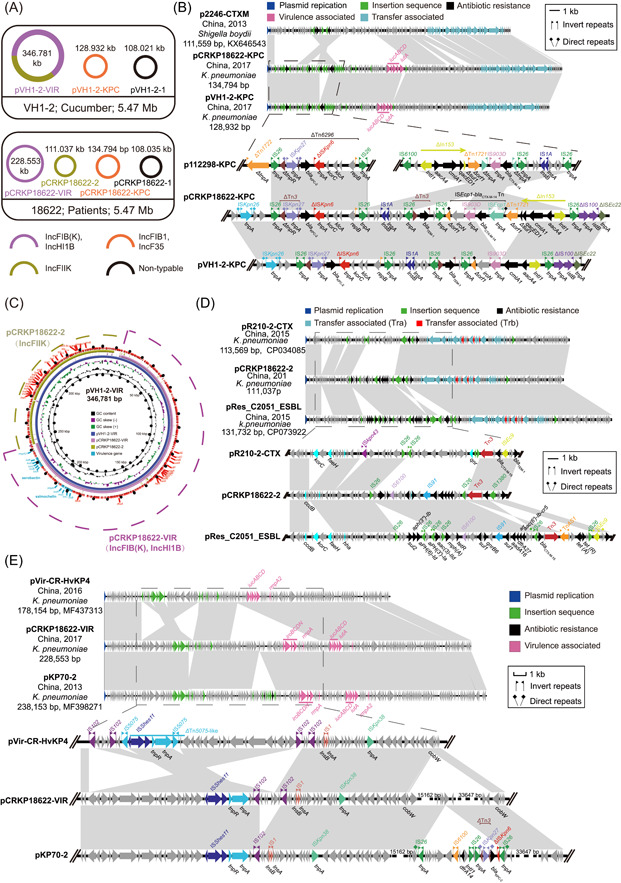
Genome sequence analysis of *Klebsiella pneumoniae* strains VH1‐2 and 18622. (A) Species, sources, and chromosome sizes of the CRKP isolates. (B) Comparison of plasmids pCRKP18622‐KPC, pVH1‐2‐KPC, and p112298‐KPC. (C) Circular comparison of plasmid pVH1‐2‐VIR, pCRKP18622‐VIR, and pCRKP18622‐2. The plasmid pVH1‐2‐VIR located at the innermost circle was used as the reference plasmid to perform sequence alignment with BLASTn by the BRIG software. Genetic regions associated with virulence are highlighted in cyan. (D) Linear alignment of complete plasmid sequences of pR210‐2‐CTX, pCRKP18622‐2, and pRes_C2051_ESBL. Genes are denoted by arrows and colored based on function classification. Shaded regions denote shared regions of homology (95% nucleotide identity). (E) Linear alignment of complete plasmid sequences of pVir‐CR‐HvKP4, pCRKP18622‐VIR, and pKP70‐2.

Whole genome sequencing (WGS) analysis of the genomes of these two strains revealed the presence of three and four plasmid types in VH1‐2 and 18622, respectively (Figure 1A). Isolate 18622 contained *bla*_KPC‐2_ on an F35:A‐:B1 type plasmid pCRKP18622‐KPC (134,794 bp, Genbank Accession No. CP027866), which carried *aac(6′)‐Ib*, *bla*_TEM‐1B_, *bla*_CTX‐M‐14_, *aac(6′)‐Ib‐cr*, *cmlA1*, and *iucABCD‐iutA*. Notably, isolate VH1‐2 from cucumber contained a highly similar *bla*_KPC‐2_‐bearing F35:A‐:B1 type plasmid. The backbone of both *bla*_KPC‐2_‐bearing plasmids was organized similar to plasmid p2246‐CTXM (Genbank Accession No. KX646543) carrying *bla*_CTX‐M‐14_ from *Shigella boydii*. The primary component of the multidrug‐resistant (MDR) region on pCRKP18622‐KPC was *bla*_KPC‐2_ and *bla*_CTX‐M‐14_. The *bla*_KPC‐2_ was located in the ΔTn*6296* that inserted at a site downstream of IS*Kpn26* in both strains 18622 and VH1‐2 and differed from the site in p112298‐KPC (Genbank Accession No. NZ_KP987215). The *bla*_CTX‐M‐14_ transposition unit was flanked by 5 bp direct repeats (DRs) at both ends, located downstream of *bla*_KPC‐2_ and inserted on a reverse ΔTn*1721* on plasmid pCRKP18622‐KPC. However, the *bla*_CTX‐M‐14_ transposition unit was truncated and further disrupted by ΔIn*153* and IS*26* elements on plasmid pVH1‐2‐KPC, resulting in the minor differences between pVH1‐2‐KPC and pCRKP18622‐KPC (Figure 1B).

Numerous virulence genes were identified on the plasmids in both strains and VH1‐2 possessed *rmpA*, *iroBCDN*, *iucABCD‐iutA*, and *rmpA2* on a 346,781 bp IncHI1B‐FIIK2‐FIB(K) plasmid designated as pVH1‐2‐VIR, which also carried ARGs for β‐lactams (*bla*_CTX‐M‐15_, *bla*_TEM‐1C_), fluoroquinolones (*qnrB6, aac(6′)‐Ib‐crcr*), aminoglycosides (*aadA16*), rifampicin (*arr‐3*), sulfonamides (*sul1*), macrolide (*mph*(A)), and trimethoprim (*dfrA27*). Interestingly, the virulence plasmid pVH1‐2‐VIR covered all the regions of both the IncFIIK resistance plasmid (pCRKP18622‐2) and the IncFIB(K)‐IncHI1B virulence plasmid (pCRKP18622‐VIR) and carried the ARGs *aadA16*, *bla*_CTX‐M‐15_, *bla*_TEM‐1C_, *aac(6′)‐Ib‐cr*, *qnrB6*, *mph(A)*, *arr‐3*, *sul1*, *dfrA27* as well as virulence genes *iucABCD‐iutA*, *iroBCDN*, *rmpA*, and *rmpA2*, respectively (Table S1). Moreover, sequence comparisons indicated that the virulence plasmid pVH1‐2‐VIR was formed by the fusion of pCRKP18622‐2 and pCRKP18622‐VIR carried by strain 18622 (Figure 1C).

The backbone of plasmids pR210‐2‐CTX (Genbank Accession No. CP034085) and pRes‐C2051‐ESBL (Genbank Accession No. CP073922) from *K. pneumoniae* in the GenBank database were similar to pCRKP18622‐2, especially for pRes‐C2051‐ESBL. Compared with pCRKP18622‐2, the plasmid pRes‐C2051‐ESBL was inserted with a 5036 bp *hp‐korc‐hp‐fesH‐hp* fragment and a 9221 bp MDR region mediated by IS*26* in two identical directions. In particular, a 10,971 bp tetracycline resistance gene region flanked by Tn*AS1* and IS*26* in two opposite directions was inserted in plasmid pRes‐C2051‐ESBL (Figure 1D). The conjugative transfer regions including *tra* and *trb* genes were identified in the plasmid pCRKP18622‐2 and pVH1‐2‐VIR (Figure 1C,D).

An identical multidrug resistance region (MRR) of 29,479 bp containing *bla*_CTX‐M‐15_, *aac (6′)‐Ib‐cr* and *qnrB6* as well as another six ARGs were found in both pVH1‐2‐VIR and pCRKP18622‐2 and both harbored virulence genes *iucABCD‐iutA*, *iroBCDN*, *rmpA*, and *rmpA2*. In the GenBank database, there was no highly similar plasmid to pVH1‐2‐VIR whereas pKP70‐2 (Genbank Accession No. MF398271) from a clinical CR‐HvKP isolate in China showed the highest similarity to pCRKP18622‐VIR in this study. The minor differences between these two plasmids were two unique regions and a segment of 3645 bp franked by two IS*102* in the same orientation was inserted in pCRKP18622‐VIR. Furthermore, an MDR region of 14,780 bp flanked by two IS*26* elements containing 8 bp DRs (CTAAAATT) in the same orientation was inserted between the backbone region in pKP70‐2, suggesting that the insertion of this MDR region into the virulence plasmid pCRKP18622‐VIR was mediated by IS*26* to form pKP70‐2 (Figure 1E).

### Prevalence of pVH‐2‐VIR‐like plasmids

**Figure 2:**
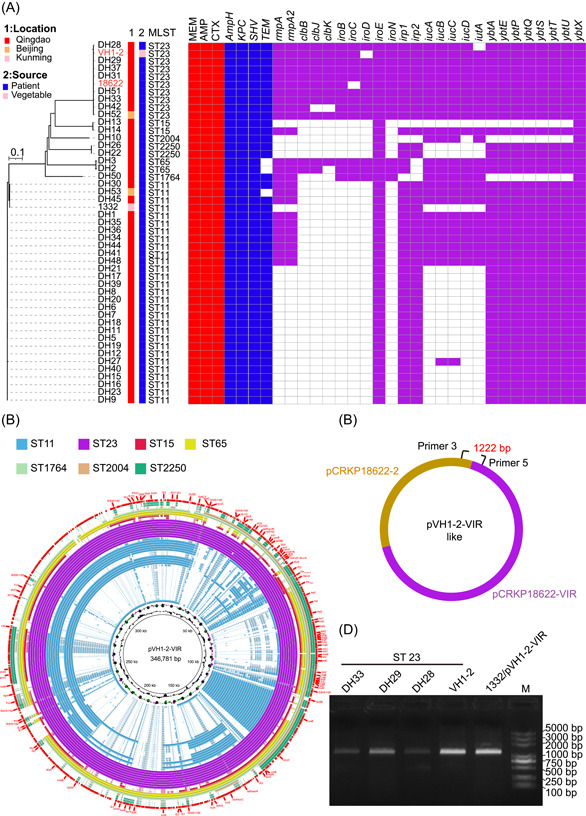
Prevalence of pVH1‐2‐VIR‐like plasmids. (A) Phylogenetic structures, MLST, antibiotic resistance phenotypes, ARGs, and virulence genes of CRKP isolates. Isolate location and sources are indicated. Red and blue squares represent positivity for resistance phenotypes and resistance genes, respectively. Purple squares represent positivity for virulence genes. AMP, ampicillin; CTX, cefotaxime; MEM, meropenem. (B) Sequence alignment of virulence plasmids harboring *rmpA* and *rmpA2* in the 23 clinical CR‐HvKP strains of different STs used in this study. The 346.781 kb virulence plasmid pVH1‐2‐VIR found in this study is used as a reference and genetic regions associated with virulence are highlighted in green. (C) Schematic depicting for investigating potential pVH1‐2‐VIR‐like plasmids. Primers 3 and 5 were used to amplify hybrid regions. (D) PCR confirmed the presence of the pVH1‐2‐VIR‐like plasmids in CRKP isolates. CRKP, carbapenem‐resistant *K. pneumoniae*.

To investigate the prevalence of such virulence plasmid, we retrospectively screened 45 clinical CRKP strains collected from Qingdao, Beijing, and Kunming in China in 2017–2018. All the 45 clinical CRKP strains that carried *bla*_KPC‐2_ and 23 (51.11%) possessed the virulence plasmids harboring *rmpA* and *rmpA2*. Both the CRKP isolates (1742 and 1743) from Beijing were CR‐HvKP, possessing *rmpA* and *rmpA2*, and 21/45 (46.67%) clinical CRKP strains were classified as CR‐HvKP. WGS analysis indicated that five different ST types were present in the 23 clinical CR‐HvKP strains, among which ST11 (10 strains) and ST23 (10 strains) were the most prevalent (Figure 2A). We also found that all ST11 and ST1764 CR‐HvKP strains lacked the *clbA‐S* virulence gene cluster, which was found in all examined ST23 and ST65 clinical CR‐HvKP strains. Of note, all 23 CR‐HvKP strains carried the gene cluster *iucABCD‐iutA*, which was also located in the virulence plasmid pVH1‐2‐VIR harboring *rmpA2*, and genes *ybtA/E/P/Q/S/T/U/X* were found in 42 clinical CRKP strains (Figure 2A).

Owing to its hybrid nature encompassing both the virulence plasmid pCRKP18622‐VIR and resistance plasmid pCRKP18622‐2, the 346.781 kb virulence plasmid pVH1‐2‐VIR was aligned to pVH1‐2‐VIR in 47 CR‐HvKP strains of varying STs. A retrospective analysis of clinical records revealed that the plasmid contigs within the ST23 strains covered nearly all regions of pVH1‐2‐VIR (Figure 2B) indicating that CR‐HvKP from patients exhibited similarity with those from ready‐to‐eat vegetables.

To further investigate the prevalence of the pVH1‐2‐VIR‐like plasmid, two pairs of primers were designed to target the hybrid region for PCR amplification and sequencing (Figure 2C). The results of the PCR amplification and sequencing confirmed that four ST23 isolates from hospital patients in Qingdao, Shandong province, China (DH33, DH29, DH28, and VH1‐2) were positive for the hybrid region (Figure 2D). This indicated that the occurrence of pVH1‐2‐VIR‐like virulence plasmids was not limited to VH1‐2 but was also present in other clinical ST23 CRKP strains.

### The pVH1‐2‐VIR plasmid contributes to hypervirulence in clinical ST11 CRKP

Given that ST11 has been the predominant CRKP type in China and the majority of ST11 CRKP strains are not hypervirulent, conjugation experiments were conducted to assess the potential hazard of the pVH1‐2‐VIR plasmid using a clinical ST11 strain as the recipient. The results of the conjugation experiments demonstrated that the pVH1‐2‐VIR plasmid was transmissible to ST11 CRKP1332 from VH1‐2 at a conjugative frequency of 2.2 ± 0.56 × 10^−6^. Of note, the transconjugant 1332 demonstrated resistance to amikacin while remaining susceptible to rifampicin whereas VH1‐2 exhibited resistance to rifampicin but remained susceptible to amikacin. MIC tests demonstrated that the acquisition of pVH1‐2‐VIR resulted in resistance to amikacin and rifampicin, indicating the successful transfer of pVH1‐2‐VIR into strain 1332. Additionally, both 1332/pVH1‐2‐VIR and 1332 exhibited comparable susceptibilities to meropenem, rifampicin, fosfomycin, gentamicin, ciprofloxacin, tetracycline, and doxycycline (Tables S2 and S3).

**Figure 3:**
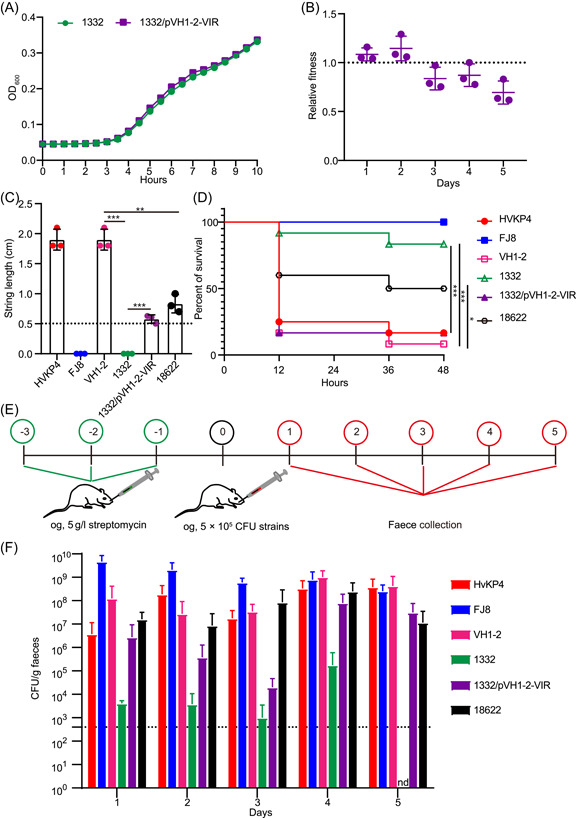
The pVH1‐2‐VIR plasmid contributes to hypervirulence to clinical ST11 CRKP. (A) Growth curve of 1332 and 1332/pVH1‐2‐VIR (*n* = 3). (B) Relative fitness of pVH1‐2‐VIR plasmid carrying clones compared with pVH1‐2‐VIR‐free clones obtained by competition assays. Bars represent normalized relative fitness after subtracting the effect of pVH1‐2‐VIR (*n* = 3). (C) String length observed in tested CRKP isolates (*n* = 3). (D) Virulence potential of CRKP isolates tested in a *Galleria mellonella* infection model. Survival of mice infected with 5 × 10^5^ CFU of each *Klebsiella pneumoniae* at 48 h is shown (*n* = 12). (E) Experimental setup for the intestinal colonization assay. (F) Colonization levels of CRKP isolates in feces at Days 1 to 5 after infection (*n* = 6). *p < 0.05; **p < 0.01; ***p < 0.001. CFU, colony‐forming unit; CRKP, carbapenem‐resistant *K. pneumoniae*; nd, not detected.

Interestingly, the growth curves of the transconjugant 1332/pVH1‐2‐VIR and donor were indistinguishable (Figure 3A). This indicated that the introduction of pVH1‐2‐VIR plasmid did not alter the normal growth of donor 1332. Moreover, since plasmids can introduce fitness costs to their hosts, we performed a quantitative evaluation of the fitness costs linked to pVH1‐2‐VIR carriage. We carried out a competition between isogenic 1332/pVH1‐2‐VIR and 1332 clones. However, the outcomes of these assays indicated that pVH1‐2‐VIR introduced only minor fitness costs (Figure 3B).

The virulence potentials of strains 18622, VH1‐2, 1332, and 1332/pVH1‐2‐VIR were also assessed through viscous string assay and the *G. mellonella* larvae survival assay. Plasmid HVKP4 was used as a hyper‐virulence control and FJ8 as a low‐virulence control^18^. We found that both FJ8 and 1332 generated viscous string <0.5 cm, indicating a low‐mucoviscous phenotype. In contrast, HVKP4, 18622, VH1‐2, and 1332/pVH1‐2‐VIR exhibited a higher degree of mucoviscosity with strings >0.5 cm compared to the low‐virulence control FJ8 (Figure 3C). The virulence potential using *G. mellonella* larvae indicated that inoculation with 5 × 10^5^ CFU of VH1‐2 strain resulted in a mortality of 91.67% at 48 h and this surpassed that of the hypervirulent control strain HVKP4 (83.33% mortality) and was significantly higher than the low‐virulence control strain FJ8 (0% mortality).

The inoculation of *G. mellonella* larvae with 5.0 × 10^5^ CFU 1332/pVH1‐2‐VIR resulted in a mortality rate of 83.33% at 48 h and was comparable to that of HVKP4 but higher than that of 1332 (12.67%, *p* < 0.001). These findings provide further evidence that the acquisition of the pVH1‐2‐VIR plasmid by the ST11 CRKP strain 1332 facilitated its transformation into a hypervirulent strain. In contrast, strain 18622 displayed a lower degree of mucoviscosity (*p* < 0.01) and mortality (50%, *p* < 0.05) compared to VH1‐2, suggesting that its virulence level may be lower than that of the VH1‐2 strain (Figure 3D).

The process of gastrointestinal colonization plays a crucial role in the development of healthcare‐associated infections caused by *K. pneumoniae* strains^19^. To assess the colonization potential of CRKP strains, a streptomycin‐treated mouse model was utilized. Interestingly, despite the seemingly low virulence of strain FJ8, its colonization level was found to be significantly high in the mouse model. Specifically, infection with either 5 × 10^5^ VH1‐2 or 1332/pVH1‐2‐VIR at 5 days postinfection (dpi) resulted in a colonization level of 4.28 × 10^8^ and 3.12 × 10^7^ CFU/g, respectively. However, CRKP strain 1332 was not detected in the feces at 5 dpi. The data indicated that the presence of a pVH1‐2‐VIR plasmid in 1332 resulted in an elevated level of colonization whereas 18622 demonstrated a slightly lower level of colonization compared to VH1‐2 (Figure 3E).

## DISCUSSION

The incidence of CR‐HvKP strains has been on the rise, particularly in China^12^, ^20^. Previous reports have indicated that the carbapenemase gene *bla*_KPC‐2_ and virulence genes *rmpA/rmpA2* were typically situated on separate plasmids in most of CR‐HvKP strains^3^, ^21^. Plasmids carrying *bla*_KPC‐2_, for example pVH1‐2‐KPC, were frequently found to be transferable^6^, ^22^. Whereas hypervirulence plasmids, for example pLVPK‐like plasmids, often lacked conjugative regions like *tra* ^20^. These findings prompted the hypothesis that hypervirulent plasmids present in clinical HvKP strains lacked conjugative properties. Consequently, the formation of CR‐HvKP was believed to primarily occur via conjugative *bla*_KPC‐2_ plasmid acquisition from HvKP strains. Although pLVPK‐like virulence plasmids were initially deemed nonconjugative, a recent investigation revealed that virulence plasmids could mobilize independently or in conjunction with a helper plasmid at a lower rate^23^. Furthermore, the nonconjugative virulence plasmid could be supplied with transfer functions from a conjugative plasmid during conjugation and mobilize at a higher frequency^24^.

Previous reports have indicated that ARGs, such as *bla*_CTX‐M_ ^11^ and *bla*_KPC‐2_ ^8^, are colocated on pLVPK‐like plasmids. However, these plasmids are devoid of conjugative regions. In contrast to previously identified virulence plasmids, a novel conjugative virulence plasmid was recently identified in *K. variicola*, which integrated a 100 kb virulence fragment into a conjugative IncFIB plasmid. This plasmid was capable of being transferred to various *Klebsiella* strains via conjugation but did not harbor any ARGs^12^. In scattered clinical CRKP strains, 11 virulence plasmids were identified that were potentially capable of conjugation and contained the transfer region of plasmid IncFII, that is, *tra* genes. In addition, most of these plasmids also harbored ARGs^25^, ^26^ while only two have been demonstrated to be transferable. Recently, a conjugative hybrid plasmid of 479 kb was reported in a clinical ST15 CR‐HvKP in China. This plasmid shared regions from a 290 kb virulence plasmid and a conjugative MDR plasmid (188 kb), both of which are present in another clinical ST15 CR‐HvKP^27^.

There have been recent increases in the incidence of foodborne illnesses caused by contaminated vegetables. This is compounded by the presence of hypervirulent MDR organisms in ready‐to‐eat vegetables and poses a significant food safety concern. The current study aims to characterize a CR‐HvKP strain associated with foodborne transmission. We identified an ST23 CR‐HvKP strain from cucumber that harbored a 347 kb conjugative hybrid virulence plasmid. This plasmid was likely formed through the recombination of a virulence plasmid (228 kb) and an IncFIIK conjugative MDR plasmid (111 kb) present in clinical ST23 CRKP strains. The virulence plasmid (pVH1‐2‐VIR) contained IncFIIK, IncFIB(K), and IncHI1B replicons as well as transfer regions *tra* and *trb*. These three types of Inc plasmids are predominantly distributed among *Enterobacteriaceae*, including *E. coli*, *K. pneumoniae*, *Shigella flexneri*, and *Salmonella enterica Typhimurium* ^28^, ^29^, ^30^. Our conjugation assays demonstrated the transferability of the pVH1‐2‐VIR plasmid to the widely prevalent clinical ST11 CRKP strain. Our conjecture is that these virulence plasmids have the potential to directly disseminate among bacterial pathogens, especially *Enterobacteriaceae*, resulting in the simultaneous expression of phenotypic carbapenem resistance and high‐level virulence. This could pose a significant threat to human health through the food chain. To the best of our knowledge, this is the first documented report of this type of particularly hypervirulent MDR plasmid, particularly in the context of the food chain.

The ST23 clonal group is associated with hypervirulence and has been identified as a prevalent ST in clinical *K. pneumoniae* across several countries including China^31^. Examination of virulence genes in CR‐HvKP revealed that all ST23 CR‐HvKP strains contained nearly all virulence genes, resulting in heightened virulence compared to other STs. This finding further supports the notion that ST23 clonal group members are highly virulent. In addition, our findings indicated that CR‐HvKP strains (specifically ST23 VH1‐2) that possess virulence plasmids containing CPS regulatory genes, exhibit greater virulence than CRKP strains lacking such plasmids. This was indicated quantitively here using *G. mellonella* assays. The hypervirulent potential of the foodborne strain VH1‐2 and its virulence plasmid transconjugant was assessed using the string test, a widely used method for identifying HvKP strains^31^. The results confirmed that VH1‐2 and its transconjugants exhibited high levels of virulence and provided additional evidence that the acquisition of a conjugative hypervirulent plasmid by ST11 1332 contributed to the observed increase in virulence.

The cocarriage of ARGs and virulence factors within a singular virulence vector can facilitate the concurrent transmission of both traits, thereby promoting the rapid emergence and dissemination of multidrug‐resistant hypervirulent *K. pneumoniae* (MDR HvKP) strains^32^. Our results of colonization assay further confirmed that the acquisition of the virulence plasmid could augment mammalian colonization, thereby posing an unprecedented threat to human health via the food chain. Notably, VH1‐2 exhibited a greater degree of virulence and colonization relative to 18622, suggesting that the fusion of the two plasmids enhanced not only virulence but also bacterial colonization. However, the underlying mechanisms remain unclear and require further investigation.

In brief, this study delineated the emergence of an infrequent conjugative MDR hypervirulent plasmid in an ST23 CR‐HvKP strain isolated from fresh cucumber. This conjugative plasmid has the potential to directly disseminate into human CRKP and contribute to augmented virulence and colonization, thereby posing an unparalleled risk to human health through the food chain. The emergence of conjugative MDR hypervirulent plasmids in pathogens found in ready‐to‐eat food is a concerning trend and it is imperative to closely monitor the prevalence of such plasmids among clinical strains and the food chain.

## MATERIALS AND METHODS

### Bacterial strains antimicrobial susceptibility testing and PFGE typing

In 2017–2018, a total of 47 isolates were obtained from inpatient sputum and cucumber samples in Qingdao (*n* = 44), Beijing (*n* = 2), and Kunming (*n* = 1), China. The agar dilution method was used to determine the minimum inhibitory concentrations (MIC) for meropenem, cefotaxime, ampicillin, fosfomycin, amikacin, ciprofloxacin, gentamicin, tetracycline, trimethoprim/sulfamethoxazole, rifampicin, and florfenicol. The results were interpreted in accordance with the Clinical and Laboratory Standards Institute (CLSI) guidelines. Broth microdilution method was used to examine the MIC of tigecycline. *E. coli* ATCC 25922 was used as a quality control strain. Clonal relationships of VH1‐2 and 18622 were investigated using PFGE of *Xba*I‐digested genomic DNA as previously described^33^.

### WGS and phylogenetic analysis

WGS was conducted on 47 selected isolates using the Illumina MiSeq system (Illumina), and the paired‐end Illumina reads were assembled using SPAdes v3.6.2^34^. ARG was identified via ResFinder 3.1 (https://cge.cbs.dtu.dk/services/ResFinder/) while transposon and insertion sequence (IS) elements were predicted using ISfinder (https://www-is.biotoul.fr). The prediction of virulence genes was facilitated with the Virulence Factor Database (http://www.mgc.ac.cn/VFs/). The genome was annotated through the Prokaryotic Genome Annotation Pipeline server. The BLASTn algorithm, integrated in BLAST ring image generator (BRIG) Version 0.95 was employed for sequence comparisons and map generation^35^. In addition, EasyFig. 2.1 (BLASTn, default setting) was utilized for the linear comparison of plasmids^36^.

### Identification of pVH1‐2‐VIR‐like plasmid

The CRKP isolates were screened for the presence of pVH1‐2‐VIR‐like hybrid plasmid using primers F3: 5′‐GCACTCCGGAATACATACTGATG‐3′, F5: 5′‐GCAAAAAGGGCGTGAACTTCAGG‐3′. The samples were run with the following PCR program: 95°C 3 min, 95°C 15 s–55°C 15 s–72°C 1 min for 30 cycles, 72°C 5 min. The presence of the targeted gene was confirmed by Sanger sequencing.

### Conjugation experiments

The conjugation experiments were conducted following previously established protocols with minor modifications^37^. Specifically, the donor VH1‐2 (ST23, rifampicin‐resistant) and recipient *K. pneumoniae* 1332 (ST11, amikacin‐resistant) were each inoculated into 5 ml of LB broth at a concentration of 10^8^ colony‐forming units (CFU)/ml and incubated at 37°C. After 6 h of liquid mating, the mixed suspension was subjected to serial dilutions and plated on selective media as follows: (1) LB agar supplemented with rifampicin (128 µg/ml) and amikacin (64 µg/ml) for the enumeration of transconjugants; (2) LB agar containing amikacin (64 µg/ml) for the calculation of recipient cells. The identity of transconjugants was confirmed by PCR assays and ERIC‐PCR^38^. Conjugative frequency was calculated as: *γ* = *T*/*R*, *T* and *R* represent the number of transconjugants and recipients per milliliter, respectively^39^. The experiment was performed in triplicate.

### Growth curves

The bacterial cells of *K. pneumoniae* 1332 and 1332/pVH1‐2‐VIR were cultured overnight and subsequently adjusted to a concentration of 5 × 10^5^ CFU/ml with LB broth. The cultures were then incubated at 37°C with shaking at 180 rpm. Growth curves were generated by measuring the optical density at 600 nm for a duration of 10 h utilizing an EnSight Multimode Microplate Reader (PerkinElmer). Prism 8 software (GraphPad) was employed to construct the growth curves.

### Competition assay

The *K. pneumoniae* 1332 and 1332/pVH1‐2‐VIR strains were cultured overnight, washed, and resuspended in LB broth. Subsequently, the cultures were combined in a 1:1 ratio and inoculated at a 1:100 dilution. The resulting mixtures were incubated at 37°C for 1, 2, 3, 4, and 5 days. Following incubation, LB agar supplemented with or without rifampicin (128 µg/ml) was utilized to plate dilutions of the bacterial mixture, enabling differentiation between the rifampicin‐resistant 1332/pVH1‐2‐VIR and 1332 strains. Relative fitness was calculated as the ratio of Malthusian parameters *W* = log_10_(1332/pVH1‐2‐VIR_end_/1332/pVH1‐2‐VIR_start_)/log_10_(1332_end_/1332_start_) as previously described^40^. Three independent competition experiments were performed to calculate the Malthusian parameters.

### String tests

The methodology for the string test was executed in accordance with the previously published protocol^5^. All CRKP isolates were cultured on agar plates supplemented with 5% sheep blood and incubated at 37°C for one night. The hyper‐mucoviscous phenotype was identified by observing the formation of a viscous string measuring greater than 5 mm in length generated by pulling a single colony upwards using a standard inoculation loop.

### The *Galleria mellonella* infection assay

The *G. mellonella* infection assay was conducted in accordance with the methodology outlined in a previous study^41^. Each group was comprised of a sample population of 12 larvae weighing 300 ± 50 mg. All CRKP isolates were cultured in LB broth and harvested during the exponential phase. Following a wash with sterile phosphate‐buffered saline (PBS), 10 μl of 5 × 10^5^ CFU bacterial cells were administered via injection into the rear left proleg of the larvae. The survival rate of the *G. mellonella* larvae was monitored and recorded at regular 12 h intervals over a period of 48 h. Survival was analyzed using the Kaplan–Meier method and implemented with Prism 8.0 (GraphPad).

### Mouse colonization assay

Female BALB/c SPF mice (8 weeks old, weighing 20 ± 2 g) were utilized for colonization assays. All mice were housed in the Laboratory Animal Center of Southern Medical University in Guangzhou, China. To overcome colonization resistance, the mice were pretreated with 200 μl streptomycin (5 g/l) via intragastric gavage for 3 consecutive days. The CRKP strains were experimentally introduced as previously described^42^. *K. pneumoniae* strains were cultured overnight, washed, and adjusted to a concentration of 5 × 10^7^ CFU/ml with sterile PBS. The mice were subjected to oral gavage with 200 µl of 5 × 10^5^ CFU bacterial cells following which their feces were collected on Days 1 to 5 post‐CRKP administration. The level of colonization was assessed by plating on LB agar with 100 μg/ml ampicillin and quantifying CRKP CFU in fecal samples. The colonization assay was conducted in accordance with the guidelines stipulated in the Guide for the Care and Use of Laboratory Animals at the Laboratory Animal Center of South China Agricultural University.

### Statistical analysis

Statistical analysis was performed using the software GraphPad Prism version 8.0. The significance of differences (*p* < 0.05) was determined using the unpaired *t* test with a two‐tailed nonparametric analysis.

## AUTHOR CONTRIBUTIONS

**Gong Li**: Investigation (lead); methodology (equal); software (equal); validation (equal); visualization (lead); writing—original draft (lead). **Ling Jia**: Data curation (equal); formal analysis (equal); methodology (equal); validation (supporting); visualization (equal). **Lei Wan**: Investigation (supporting). **Lijuan Xia**: Investigation (supporting); methodology (supporting). **Ang Gao**: Methodology (supporting). **Runshi Yang**: Investigation (supporting); validation (supporting); visualization (supporting). **Ruanyang Sun**: Methodology (supporting). **Minge Wang**: Data curation (supporting); formal analysis (supporting); validation (supporting); visualization (supporting). **Juan Du**: Formal analysis (supporting); validation (supporting). **Xinlei Lian**: Software (supporting). **Rongmin Zhang**: Software (supporting); visualization (supporting). **Liangxing Fang**: Writing—review and editing (supporting). **Xiaoping Liao**: Supervision (supporting). **Yahong Liu**: Funding acquisition (equal); project administration (equal); supervision (lead). **Bao‐Tao Liu**: Conceptualization (equal); data curation (equal); funding acquisition (supporting); methodology (supporting); project administration (supporting); resources (equal); validation (lead); writing—original draft (equal); writing—review and editing (equal). **Jian Sun**: Conceptualization (equal); funding acquisition (equal); project administration (equal); resources (supporting); validation (equal); writing—original draft (equal); writing—review and editing (lead).

## ETHICS STATEMENT

Animal experimentation was approved by the Ethics Committee of the Laboratory Animal Center of South China Agricultural University (Guangzhou, China), approval number 2022C16.

## CONFLICT OF INTERESTS

The authors declare no conflict of interests.

## Supporting information

Supporting information.

Supporting information.

## Data Availability

All sequencing data have been deposited in GenBank under BioProject accession number PRJNA885955.
