# Supplementary material for: Acquisition of a novel conjugative multidrug‐resistant hypervirulent plasmid leads to hypervirulence in clinical carbapenem‐resistant Klebsiella pneumoniae strains
Source: mLife. 2023 Sep 28;2(3):317–27. doi: 10.1002/mlf2.12086 (PMC10989919; doi:10.1002/mlf2.12086)
Supplement: Supplementary file 1 — Supporting information. [file MLF2-2-317-s002.docx]

**Supplementary data**

**Fig S1. MLST and PFGE-based dendrogram of the *K. pneumoniae* isolates used in this study.**

**Table S1. Antibiotic resistance genes and virulence genes carried by VH1-2 and 18622.**

**Table S2. Characteristics of three CRKP isolates.**

**Table S3. Antibiotic resistance characteristics of VH1-2, 1332 and their corresponding transconjugants.**


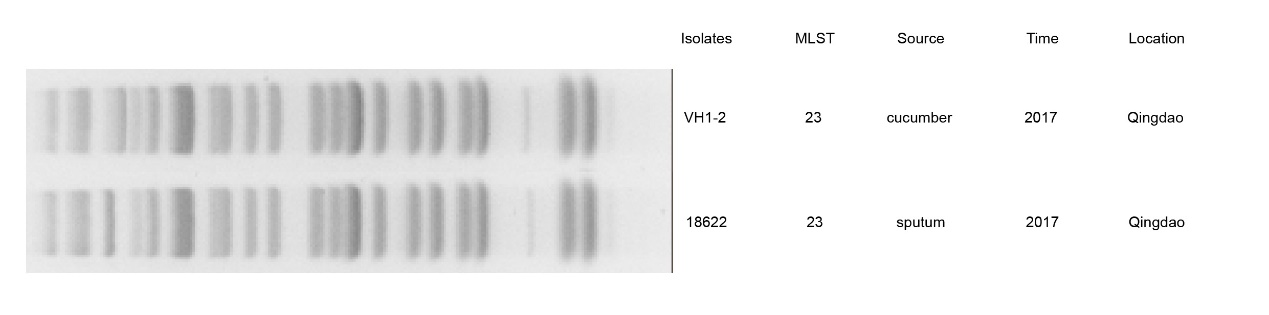


**Fig S1.** MLST and PFGE-based dendrogram of the *K. pneumoniae* isolates used in this study.

**Table S1. Antibiotic resistance genes and virulence genes carried by VH1-2 and 18622.**

|  | **Sizes (bp)** | **ST or Plasmid replicons** | **ARGs** | **Virulence genes** |
| --- | --- | --- | --- | --- |
| **VH1-2** |  |  |  |  |
| chromosome | 5,468,126 | ST23 | *bla*_SHV-190_, *oqxAB*, *fosA* | *clbB*, *clbJ*, *clbK*, *irp1*, *irp2* |
| pVH1-2-1 | 108,021 | Non-typable | NA | NA |
| pVH1-2-KPC | 128,932 | IncFIB1, IncF35 | *aac(6')-Ib*, *bla*_TEM-1B_, *bla*_KPC-2_, *aac(6')-Ib-cr*, *cmlA1* | *iucA-45*, *iucB-24*, *iucC-33*, *iucD-35*, *iutA-61* |
| pVH1-2-VIR | 346,781 | IncHI1B, IncFIIK2, IncFIB(K) | *aadA16*, *aac(6')-Ib-cr*, *qnrB6*, *bla*_CTX-M-15_, *bla*_TEM-1C_, *mph(A)*, *arr-3*, *sul1*, *dfrA27* | *iucA-1*, *iucB-1*, *iucC-1*, *iucD-1*, *iutA-1*, *iroB-1*, *iroC-4*, *iroD-1*, *iroN-1*, *rmpA-2*, *rmpA2-5* |
| **18622** |  |  |  |  |
| chromosome | 5,467,901 | ST23 | *bla*_SHV-190_, *oqxAB*, *fosA* | *clbB*, *clbJ*, *clbK*, *irp1*, *irp2* |
| pCRKP18622-1 | 108,035 | Non-typable | NA | NA |
| pCRKP18622-2 | 111,037 | IncFIIK | *aadA16*, *bla*_CTX-M-15_, *bla*_TEM-1C_, *aac(6')-Ib-cr*, *qnrB6*, *mph(A)*, *arr-3*, *sul1*, *dfrA27* | NA |
| pCRKP18622-KPC | 134,794 | IncFIB1, IncF35 | *aac(6')-Ib*, *bla*_TEM-1B_, *bla*_KPC-2_, *bla*_CTX-M-14_, *aac(6')-Ib-cr*, *cmlA1* | *iucA-45*, *iucB-24*, *iucC-33*, *iucD-35*, *iutA-61* |
| pCRKP18622-VIR | 228,553 | IncFIB(K), IncHI1B | NA | *iucA-1*, *iucB-1*, *iucC-1*, *iucD-1*, *iutA-1*, *iroB-1*, *iroC-4*, *iroD-1*, *iroN-1*, *rmpA-2*, *rmpA2-5* |

**Table S2. Characteristics of three CRKP isolates.**

| **Organism and isolate**  **(ST types)#** | **Source** | **Market/City** | **Resistance phenotypes** |
| --- | --- | --- | --- |
| VH1-2  (ST23) | Cucumber | QingDao | MEM, AMP, CTX, FOS, CIP, SXT, RIF |
| 18622（ST23） | Sputum | QingDao | MEM, AMP, CTX, FOS, CIP, SXT, RIF |
| 1332  （ST11） | [patient](C:/Users/Administrator/AppData/Local/youdao/dict/Application/7.3.0.0817/resultui/dict/?keyword=patient) | KunMing | AMK, GEN, MEM, AMP, CTX, FOS, FFC, CETE, SXT |

AMK, amikacin; GEN, gentamicin; MEM, meropenem; AMP, ampicillin; CTX, cefotaxime; FOS, fosfomycin; CIP, ciprofloxacin; FFC, florfenicol; CETE, chlortetracycline; SXT, sulfamethoxazole/dimethyl sulfoxide；RIF, Rifampicin.

**Table S3. Antibiotic resistance characteristics of VH1-2, 1332 and their corresponding transconjugant 1332/pVH1-2-VIR.**

| **Antibiotics** | **MIC (μg/mL)** | | |
| --- | --- | --- | --- |
|  | **VH1-2** | **1332** | **1332/pVH1-2-VIR** |
| MEM | >16 | >16 | >16 |
| RIF | >256 | 8 | >256 |
| FOS | 256 | >256 | >256 |
| AMK | 16 | >256 | >256 |
| GEN | 2 | >256 | >256 |
| CIP | 2 | >256 | >256 |
| TET | 1 | 8 | 8 |
| DOX | 1 | 8 | 8 |
| AMP | >256 | >256 | >256 |
| FFC | 4 | 8 | 16 |

MEM, meropenem; RIF, Rifampicin; FOS, fosfomycin; AMK, Amikacin; GEN, gentamicin; CIP, ciprofloxacin; TET, tetracycline; doxycycline; AMK, amikacin; FFC, florfenicol.
